# Efficacy of intranasal administration of dexmedetomidine in combination with midazolam for sedation in infant with cleft lip and palate undergoing CT scan: a randomized controlled trial

**DOI:** 10.1186/s12871-023-02397-2

**Published:** 2024-01-02

**Authors:** Xiaodong Wang, Lian Ma, Xudong Yang, Yi Zhou, Xiang Zhang, Fang Han

**Affiliations:** 1 Department of Anesthesiology, Peking University Hospital of Stomatology, NO. 22 Zhongguancun South Avenue, Beijing, 100081 China; 2 Department of Oral and Maxillofacial Surgery, Peking University School and Hospital of Stomatology, National Clinical Research Center for Oral Diseases, Beijing, China

**Keywords:** Intranasal sedation, Dexmedetomidine, Midazolam, Cleft lip and palate

## Abstract

**Background:**

There is a great challenge to sedation for infants with cleft lip and palate undergoing CT scan, because there is the younger age and no consensus on the type, dosage, and route of drug administration.

**Objective:**

This study aimed to evaluate the efficacy of intranasal administration of dexmedetomidine combined with midazolam as a sedative option for infants with cleft lip and palate under imaging procedures.

**Methods:**

Infants scheduled for cleft lip and palate repair surgery were randomly assigned to the IND group (intranasal dexmedetomidine 2 µg/kg alone) and the INDM group (intranasal dexmedetomidine 2 µg/kg combined with midazolam 0.05 mg/kg). The primary outcome was the proportion of infants underwent successful computed tomography (CT) scans under intranasal sedation. The secondary outcomes included onset time and duration of sedation, recovery time, Ramsay sedation scale, hemodynamic parameters during sedation, and adverse events. Data analyses involved the unpaired t-test, the repeated-measures analysis of variance test, and the continuity correction χ2 test.

**Results:**

One hundred five infants were included in the analysis. The proportion of infants underwent successful CT scans under sedation was significantly greater in the INDM group than in the IND group (47 [95.9%] vs. 45 [80.4%], *p* = 0.016). Additionally, the INDM group had a shorter onset time and a longer duration of sedation statistically (12 [8.5, 17] min vs. 16 [12, 20] min, *p* = 0.001; 80 [63.6, 92.5] min vs. 68.5 [38, 89] min, *p* = 0.014, respectively), and their recovery time was significantly longer (43 [30, 59.5] min vs. 31.5 [20.5, 53.5] min, *p* = 0.006). The difference in Ramsay sedation scale values 20 min after administration was statistically significant between the groups. No statistically significant difference was found between the groups in changes in heart rate and respiratory rate.

**Conclusion:**

Intranasal administration of dexmedetomidine in combination with midazolam resulted in higher sedation success in comparison with sole dexmedetomidine. However, it has a relatively prolonged duration of sedation and recovery time.

**Trial registration:**

ChiCTR2100049122, Clinical trial first registration date: 21/07/2021.

## Introduction

Perioperative anxiety and agitation are common in pediatric patients in hospitals. Although some procedures are painless, such as computed tomography (CT) and magnetic resonance imaging (MRI). Pediatric patients still experience discomfort and distress when separated from their parents and demonstrate uncooperativeness and movement [1]. The use of sedatives may help to reduce anxiety, alleviate the fear of being separated from their parents and minimize any delay in treatment [2]. Clinically, there are many options for sedatives, such as chloral hydrate, midazolam, dexmedetomidine, and ketamine [3]. Some sedatives can also be administered by several routes, such as oral, intravenous, and intranasal [4, 5]. Many sedatives have achieved high success rates. However, although sedation is effective, it also has some disadvantages, such as the need for monitored anesthesia care, the need for an anesthesia machine, and a bad induction experience [6, 7].

It should be noted that midazolam is commonly used as a sedative via several types of routes [3, 8]. Moreover, the intranasal route has significant advantages, such as rapid onset and painlessness when compared with oral and intravenous routes. However, it was found that intranasal administration of midazolam could irritate the nasal mucosa and cause agitation [6]. Dexmedetomidine (DEX) is another sedative that has been used extensively, which is a potent, selective alpha2 agonist, and its intranasal doses vary from 0.5 to 4 μg/kg [9, 10]. There have been reports of intranasal administration of DEX causing hemodynamic instability, such as bradycardia, which should be avoided in pediatric patients [11]. Furthermore, excessive doses may lead to inadequate mucosal absorption, influx into the pharyngeal cavity, and inconsistent dose and sedative effects [12]. Considering the disadvantages of midazolam and dexmedetomidine, some studies have reported the combination of the two sedatives to optimize the sedative effect. However, these studies involved the administration of dexmedetomidine intranasally plus midazolam orally [13, 14]. In addition, this type of combination was more suitable for older children than younger children because younger children tend to be uncooperative when on an oral medication, which can cause irritation and coughing.

Children with cleft lip and palate usually undergo repair surgery within 8 to 18 months because speech training is required early. With the development of surgical techniques, more children need to undergo CT scan preoperatively to analyze their craniofacial morphology. Because of the younger age of this patient group, CT scans need to be performed under sedation. It was suggested that dexmedetomidine could be used for Pierre Robin sequence (PRS) who underwent CT scan, which PRS was very common in infants with cleft lip and palate [15]. However, the use of sedative in combination for children with cleft lip and palate was rare. Given that many studies focused on the use of sedatives in combination by multiple routes and older pediatric population for imaging procedures [8, 13, 14]. The concern in this study was the use of non-invasive intranasal sedatives in combination for younger infants with cleft lip and palate. Therefore, this clinical trial aimed to evaluate the efficacy and safety of intranasal administration of dexmedetomidine in combination with midazolam as a sedative option for children with cleft lip and palate under CT scans. We hypothesized that a greater proportion of children can undergo successful CT scans under sedation by intranasal administration of dexmedetomidine in combination with midazolam.

## Materials and methods

### Study design and randomization

This study was a single-center, prospective, randomized, controlled double-blind trial, which was approved by the Peking University Hospital of Stomatology Ethics Committee (PKUSSIRB-202059188) and registered with the Chinese Clinical Trial Registry (ChiCTR2100049122). Written informed consent was obtained from the participants’ parents. The subjects of the study were patients who required cleft lip and palate repair surgery at the Peking University Hospital of Stomatology between December 2020 and March 2022. The inclusion criteria were as follows: (1) healthy systemic patients (American Society of Anesthesiologists Classifications I–II) with no other comorbidities and an age range of 1–36 months; (2) children scheduled for cleft lip and palate repair surgery requiring CT scan. The exclusion criteria were (1) congenital heart disease; (2) upper respiratory infection, pneumonia, and asthma attack within 2 weeks; (3) history of sleep apnea; (4) a refusal of participation from patients' guardians.

Randomization was performed by an independent statistician, and random numbers generated with SAS 8.0 (statistical program) were used to assign participants randomly (1:1) to two groups. One group received intranasal DEX (control group, the IND group), and the other group received intranasal DEX combined with midazolam (study group, the INDM group). The participant assignment codes were kept in sealed envelopes. Before surgery, these envelopes were provided to an inpatient pharmacist who was not involved in patient care. The allocation of the drugs was performed by this pharmacist. DEX at a concentration of 100 µg/mL and midazolam at a concentration of 5 mg/mL were used. The volumes of DEX and midazolam were 2 µg/kg and 0.05 mg/kg, respectively. The study drugs were prepared with dilution in 1-cm^3^ syringes to 0.5 mL. The syringes were delivered to an anesthesiologist in charge of intranasal administration. The patients’ parents, the attending anesthesiologist, the surgeons, and the data collection staff were blinded to the group assignment.

### Study procedures

All patients underwent routine preoperative evaluation and laboratory tests according to the hospital’s standard protocol. Routine monitoring included electrocardiography, pulse oximetry (SpO_2_), heart rate (HR), and respiratory rate (RR). The medication was administered into the two nostrils using the Intranasal Mucosal Atomization Device (Teleflex Incorporated, Wayne, PA, USA). At the end of the intranasal administration of sedatives, all patients were observed for 30 min before the CT scan, and the routine monitoring was continued during the CT scan. After the imaging procedure, patients were admitted to the post-anesthesia care unit and returned to the ward after their Aldrete score reached 9.

If their Ramsay score did not reach 5 within 30 min after intranasal administration, or there is physical movement during the CT scan, then inhalation anesthesia (sevoflurane) is needed in the radiology department. Patients were induced with 8% sevoflurane in 100% oxygen at 8 L/min, and after they fall asleep, their inhalation concentration was adjusted to 2% in 100% oxygen at 2 L/min. The patients were then transferred to the post-anesthesia care unit for a 1-h observation in the presence of one parent.

### Data collection

The proportion of children who underwent successful CT scans after a single intranasal administration was recorded. The sedation was evaluated using the Ramsay sedation scale (RSS) scores, whereby scores of 1 and 6 correspond to dangerous agitation and unarousable, respectively. The onset time of sedation was set from the beginning of the administration to an RSS score of 5, and the duration of sedation was the time from the onset of sedation to an RSS score of 2. The recovery time was the time from the end of the CT scan to the Aldrete score of at least 9 points. The hemodynamic parameters (HR and RR) and RSS during sedation were measured and recorded at intervals of 10 min within 50 min after administration. The adverse events during sedation were recorded, which included SpO_2_ < 90% lasting for 15 s, RR < 12 bpm, and bradycardia (HR < 70 bpm).

The primary outcome was the proportion of children who underwent successful CT scans under intranasal sedation. The secondary outcomes were the onset time of sedation, duration of sedation, recovery time, RSS, hemodynamic parameters during sedation, and adverse events.

### Statistical analysis

The sample size was calculated based on a previous study [16]. The proportion of children who underwent successful MRI scans was 60% when administered with DEX 2 μg/kg. The sample size was calculated assuming minimum success rate with a difference of 20% in success rate being considered clinically significant. Hence, to detect difference of 20% in the proportion of children who underwent successful CT scans with DEX combined with midazolam (i.e., minimum success rate of 80% with DEX combined with midazolam) with equal allocation. Therefore, in this study, 80% power was considered. To detect differences, it was necessary to include 45 patients per group with an alpha risk of 2.5% and a beta risk of 20% in a two-tailed comparison. To compensate for approximately 10% of dropouts during the study period, 50 patients were enrolled in each group.

Statistical analysis was performed using IBM SPSS STATISTICS 25 software (SPSS Inc., Chicago, IL, USA). The values are expressed as mean ± standard deviation (SE), median values (interquartile range), numbers, and percentages. Continuous variables with normal distribution were analyzed using the unpaired *t*-test. Categorical variables were analyzed using the continuity correction *χ*^2^ test or Fisher’s exact test. The repeated-measures variables were analyzed using the repeated-measures analysis of variance test.

## Results

### Subject characteristics

**Fig. 1:**
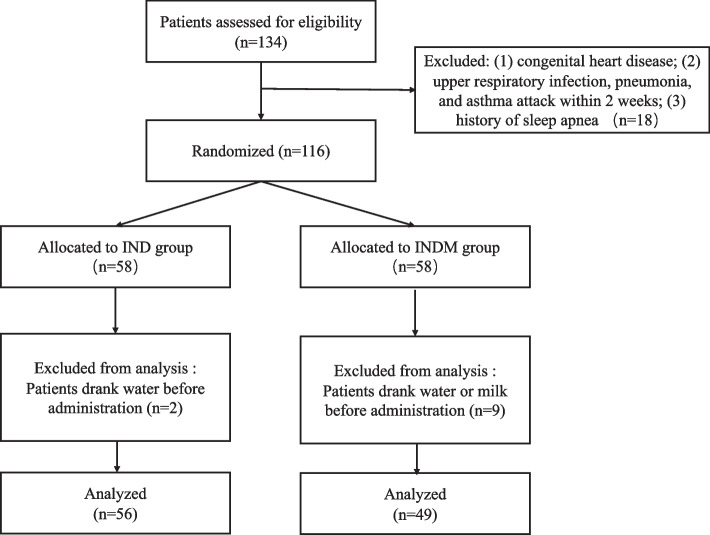
Flow diagram of patients through the trial

Two patients from the IND group were excluded because they drank water 1 h before administration. Therefore, data from a total of 105 patients were analyzed (Fig. 1). The demographic variables were not significantly different between the groups (Table 1).

**Table 1:** Demographics of pediatric patients

	IND group (*n* = 56)	INDM group (*n* = 49)	*P* value
Age, months	10.3 ± 6.8	10.4 ± 9.5	0.958
Sex (male/female)	29/27	17/32	0.078
Weight (kg)	9.1 ± 1.8	8.9 ± 1.4	0.533
Kinds of cleft (lip/palate)	3/53	3/46	0.866
Duration of CT (min)	2.2 ± 1.0	1.9 ± 0.9	0.931

Values are shown as means ± SD, and numbers of patients (n)

No significant differences were observed between the two groups, *SD* standard difference, *IND* intranasal administration of dexmedetomidine alone, *INDM* intranasal administration of dexmedetomidine in combination with midazolam

### Efficacy of sedation

There was a significantly higher proportion of children who underwent successful CT scans in the INDM group than in the IND group (47 [95.9%] vs. 45 [80.4%], *p* = 0.016) (Table 2). The onset time of sedation was significantly shorter in the INDM group than in the IND group (12 [8.5, 17] min vs. 16 [12, 20] min, *p* = 0.001). The duration of sedation was more than 50 min in both groups, whereas the duration of sedation was significantly longer in the INDM group than in the IND group (80 [63.6, 92.5] min vs. 68.5 [38, 89] min, *p* = 0.014). Furthermore, the recovery time was significantly comparable between the groups: in the INDM group, the children needed a longer time to wake up (43 [30, 59.5] min vs. 31.5 [20.5, 53.5], *p* = 0.006) (Table 3).

**Table 2:** Proportion of CT scan successfully under intranasal sedation for groups

	CT scan successfully		*P* value	RR	95%CI	
	YES (n [%])	NO (n [%])			min	max
IND group (*n* = 56)	45(80.4%)	11(19.6%)	0.016	5.744	1.206	27.367
INDM group (*n* = 49)	47(95.9%)	2(4.1%)				

Values are shown as numbers of patients (n), and percentages (%)

*IND* intranasal administration of dexmedetomidine alone, *INDM* intranasal administration of dexmedetomidine in combination with midazolam

**Table 3:** Sedation variables for groups

	IND Group (*n* = 56)	INDM Group (*n* = 49)	*P* value
The onset of sedation (min)	16 [12, 20]	12 [8.5, 17]	0.001
The duration of sedation (min)	68.5 [38, 89]	80 [63.5, 92.5]	0.014
The recovery time (min)	31.5 [20.5, 53.5]	43 [30, 59.5]	0.006

Values are shown as median (interquartile range)

*IND* intranasal administration of dexmedetomidine alone, *INDM* intranasal administration of dexmedetomidine in combination with midazolam

### Hemodynamic and RSS parameters

**Fig. 2:**
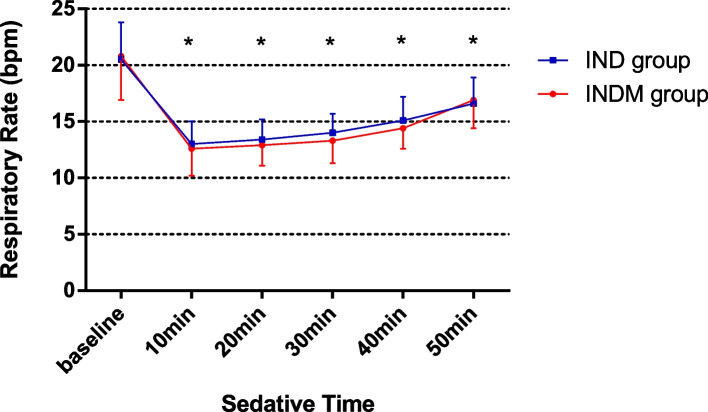
Changes in respiratory rate in both groups at six measurement points (*P < 0.05 vs baseline)

**Fig. 3:**
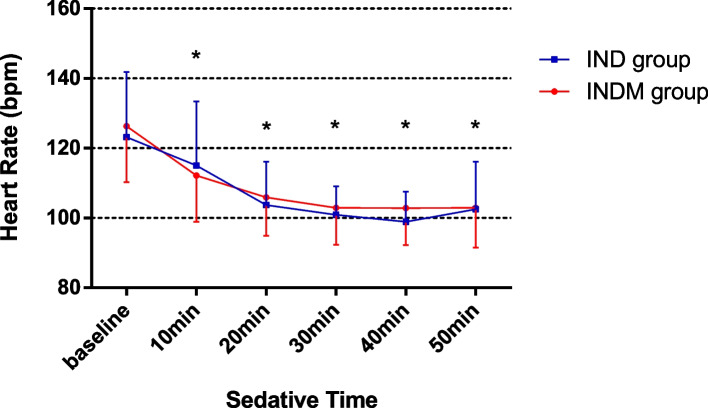
Changes in heart rate in both groups at six measurement points (*P < 0.05 vs baseline)

It was found that HR and RR after intranasal administration were significantly lower than baseline HR and RR. However, no statistically significant difference was found between the groups in the HR and RR changes at the 10th, 20th, 30th, 40th, and 50th min (Figs. 2 and 3). None of the patients experienced hypoxemia (SPO_2_ < 90%).

Although the RSS values in the IND group were lower than those in the INDM group at the 10th min, there was no statistical significance between the groups (4 [2.5, 6] vs. 3 [2, 4], *p* = 0.067). The RSS values at the 20th min were significantly different in both groups (6 [6, 6] vs. 6 [5.25, 6], *p* = 0.015). The RSS values at the 30th, 40th, and 50th min in both groups were not significantly comparable (Table 4). No adverse events were observed during the sedation procedure.

**Table 4:** Ramsay sedation scale for groups

	10 min	20 min	30 min	40 min	50 min
IND group (*n* = 56)	3 [2, 4]	6 [5.25, 6]	6 [6]	6 [6]	6 [6]
INDM group (*n* = 49)	4 [2.5, 6]	6 [6]	6 [6]	6 [6]	6 [6]
P value	0.067	0.015	0.212	0.462	0.742

Values are shown as median (interquartile range)

*IND* intranasal administration of dexmedetomidine alone, *INDM* intranasal administration of dexmedetomidine in combination with midazolam

## Discussion

The findings of this prospective, randomized, controlled double-blind trial were that, compared with sole intranasal dexmedetomidine, intranasal administration of dexmedetomidine combined with midazolam resulted in a high proportion of children who underwent successful CT scans. Furthermore, the efficacy of sedation was enhanced when dexmedetomidine was combined with midazolam.

At present, CT imaging is an extensively performed diagnostic modality in the pediatric population. However, because of the noise and tube narrowness in CT imaging, deep sedation is required in children. Intranasal dexmedetomidine appears to be a reasonable option as stated in the literature [5]. First, intranasal administration is a less invasive and manageable approach to sedative premedication in children. Second, intranasal dexmedetomidine exerts a natural sleep sedative effect, and with the help of an atomizer, the bioavailability has been reported to be 83.8% in children [17]. However, because of safety issues in younger children, the use of sole dexmedetomidine at low concentrations is adopted, which tends to have several disadvantages, such as slow onset and a certain probability of imaging failure [18] and slow onset. Therefore, to pursue a higher percentage of successful CT scans and a faster onset of action, the sedatives in combination was adopted in this study. The results indicated that after intranasal administration of a dose of 2 µg/kg of dexmedetomidine combined with 0.05 mg/kg of midazolam, the proportion of children who underwent successful CT scans was 95.9%, compared with 80.4% of children with intranasal dexmedetomidine alone. In addition, the combination use of sedatives accelerated the onset time and prolonged the duration. The above results of this study seemed to bring some convenience in the daily routine of a busy clinical setting. However, it must be pointed out that the patients involved in this study were inpatient, and hospitals should have facilities for safe recovery of the affected patients.

Undoubtedly, the efficacy of using some sedatives in combination with dexmedetomidine is better. A study by Gu H et al. revealed that oral midazolam (0.2 mg/kg) with intranasal dexmedetomidine (2–3 μg/kg) might produce an excellent sedative effect for children undergoing MRI [14]. However, considering the younger age in our study, we opted to administer midazolam intranasally rather than orally because coughing and irritation may result in uncooperativeness. In recent years, a few studies have reported that in addition to midazolam, other intranasal sedatives such as intranasal ketamine could enhance sedation induced by intranasal dexmedetomidine in young children [19, 20], and there were no adverse events in the trials. However, different reports recorded that the incidence of vomiting and emergence of agitation was identical to that in intravenous ketamine [21, 22]. Moreover, recently, ketamine has been implicated in adverse neurodevelopmental outcomes in infants [23], which should be best avoided as far as possible. Therefore, we believe that the choice of intranasal midazolam in combination with dexmedetomidine is more reasonable.

In this study, a group of young pediatric patients with cleft lip and palate were selected, and with an average age of 10 months, the participants in the present study were younger than the children in previous studies [10, 19]. Pediatric patients with cleft lip and palate are a group of more special children; their physiological status is different from that of ordinary children, such as congenital maxillofacial deformities resulting in worse growth and development, coexisting with some syndromes that cause airway obstruction [24]. Considering the aforementioned information, the ideal premedication for cleft lip and palate pediatric procedures and the most appropriate dose for children deserve further exploration. It was presumed that younger children require larger bolus doses of dexmedetomidine to achieve satisfactory sedation due to pharmacokinetic factors [25]. Yuen et al. used intranasal dexmedetomidine as a pre-induction sedative at a dose of either 1 or 2 μg/kg for 116 randomized children who were between 1 and 8 years old. The results indicated that the difference in sedation between the two doses was more obvious in children who are between 5 and 8 years old than in those who are 1–4 years old [26]. Therefore, it was observed that the pharmacokinetics and pharmacodynamics were different between young and older children. Based on this finding, it is supposed that younger children should be administered larger doses of sedatives. However, we suggest that clinicians should prioritize safety for sedation in this young population by using combinations of low-dose sedatives. In this study, no complications, such as nausea/vomiting, airway obstruction, oxygen desaturation, and bradycardia, were encountered in any of the children during the procedure, which means that a low dose (2 μg/kg) of dexmedetomidine combined with midazolam (0.05 mg/kg) by intranasal administration is suitable for this patient group.

Notably, we used a mucosal atomization device to improve bioavailability while reducing the amount of the drug introduced into the gastrointestinal tract, which is particularly important for nasal sedation in children with cleft lip and palate. The reason is that the nasal and pharyngeal passages of this type of pediatric patients are relatively open, so more fluid might flow into the gastrointestinal tract if the mucosal atomization device is not selected. Another noteworthy point is that we controlled intranasal drug volume to a total of 0.5 mL, which could have a pharmacokinetic impact [27]. Concentrated medications in small volumes (0.2–0.3 mL per nostril) are ideal, whereas volumes of more than 1 mL per nostril are not reliably absorbed and may overflow into the nasal cavity. In this study, because of blinding and the combination of sedatives, we maintained the volume at 0.5 mL, which was approximately 0.25 mL per nostril.

There are several shortcomings to our study. First, according to the latest pharmacokinetics study, the median time for intranasal dexmedetomidine to accomplish peak concentration was 37 min. In addition, the maximal sedative effect was perceived to be 45 min after dexmedetomidine administration. However, in this study, we elected to administer premedication 30 min before the CT scan on the basis of previous studies and our clinical practice. This might cause patients to awaken during the CT scan. Second, the dose–response relationship of the midazolam–dexmedetomidine combination as a premedication treatment is not the main focus of this study; thus, the dose of midazolam (0.05 mg/kg) and dexmedetomidine (2 μg/kg) has been standardized on the basis of our routine clinical practice and previous studies. Hence, larger studies should be performed to evaluate the optimal dose of combined sedatives. Third, although there were benefits of this type of sedation, such as non-invasiveness and convenience for clinical procedures, it was worth discussing in the future that other economical and convenient sedative methods were more suitable for non-hospitalized infants. Forth, the study was performed in a single clinical center with a small sample size, which not only limited the ability to detect potentially significant associations but also rendered the findings inapplicable to other clinical settings.

In conclusion, this study demonstrated intranasal administration of dexmedetomidine in combination with midazolam was an effective and safe alternative for sedation in infants with cleft lip and palate undergoing CT. In addition, it resulted in higher sedation success in comparison with sole dexmedetomidine. However, it has a relatively prolonged duration of sedation and recovery time.

## Data Availability

The data that support the findings of this study are available from the corresponding author upon reasonable request.
